# Dlgap1 knockout mice exhibit alterations of the postsynaptic density and selective reductions in sociability

**DOI:** 10.1038/s41598-018-20610-y

**Published:** 2018-02-02

**Authors:** M. P. Coba, M. J. Ramaker, E. V. Ho, S. L. Thompson, N. H. Komiyama, S. G. N. Grant, J. A. Knowles, S. C. Dulawa

**Affiliations:** 1 Department of Psychiatry, University of California, San Diego, USA; 2 Committee on Neurobiology, The University of Chicago, Chicago, USA; 3 Department of Psychiatry and the Behavioral Sciences, Zilkha Neurogenetic Institute, Keck School of Medicine, University of Southern California, Los Angeles, USA; 4 Genes to Cognition Program, Centre for Clinical Brain Sciences, Edinburgh University, Edinburgh, Scotland

## Abstract

The scaffold protein DLGAP1 is localized at the post-synaptic density (PSD) of glutamatergic neurons and is a component of supramolecular protein complexes organized by PSD95. Gain-of-function variants of *DLGAP1* have been associated with obsessive-compulsive disorder (OCD), while haploinsufficient variants have been linked to autism spectrum disorder (ASD) and schizophrenia in human genetic studies. We tested male and female *Dlgap1* wild type (WT), heterozygous (HT), and knockout (KO) mice in a battery of behavioral tests: open field, dig, splash, prepulse inhibition, forced swim, nest building, social approach, and sucrose preference. We also used biochemical approaches to examine the role of DLGAP1 in the organization of PSD protein complexes. *Dlgap1* KO mice were most notable for disruption of protein interactions in the PSD, and deficits in sociability. Other behavioral measures were largely unaffected. Our data suggest that *Dlgap1* knockout leads to PSD disruption and reduced sociability, consistent with reports of *DLGAP1* haploinsufficient variants in schizophrenia and ASD.

## Introduction

The Disks Large Associated Protein 1 gene encodes the protein DLGAP1 (also known as GKAP or SAPAP1), which localizes at the postsynaptic density (PSD)^1–7^. Genetic variants of *DLGAP1* and abnormalities of the PSD have been associated with neuropsychiatric disorders including schizophrenia^8^, autism spectrum disorder (ASD)^9^, and obsessive-compulsive disorder (OCD)^10^. For example, the case-control portion of the first Genome Wide Association Study (GWAS) for OCD found that the two single nucleotide polymorphisms (SNPs) with the lowest P-values were both located within an intron of *DLGAP1*^11^. Furthermore, a 63 kb duplication of four exomes of *DLGAP1* was observed in a sib-pair with pediatric OCD, and other comorbid disorders^12^. Conversely, a *de novo* deletion CNV of *DLGAP1*^13^, and a number of rare protein-altering variants, including missense and stop variants, have also been identified in individuals with schizophrenia^14,15^. Furthermore, ASD patients show an increased incidence of carrying non-synonymous variants of *DLGAP1*^9^. Rodent studies have shown that DLGAP1 protein complexes isolated from the hippocampal CA1 region are enriched in proteins considered risk factors for schizophrenia and ASD^16,17^. Thus, emerging data suggests that genetic variants increasing *DLGAP1* function may predispose to OCD, while variants decreasing *DLGAP1* function might predispose to schizophrenia or ASD.

The postsynaptic terminal of excitatory synapses is characterized by an electron-dense accumulation of protein, called the Post Synaptic Density (PSD). The proteome of the PSD has been extensively characterized in vertebrates (vPSD), which has a conserved set of ~1000 proteins^2^. All PSD proteins are physically organized into a hierarchy of complexes and supercomplexes (complexes of complexes)^17–19^. DLGAP1 is a scaffold component of 1.5 MDa supercomplexes, which also includes a number of different scaffolds such as Dlg1-4, Shank1-3 and also includes N-methyl-D-aspartate (NMDA) receptors, ion channels and signaling proteins^5,19,20^. Mutations in PSD95 (Dlg4) and other proteins in these supercomplexes alter the capacity of synapses to detect and respond to patterns of neural activity, control of short- and long-term synaptic strength, and modulate innate and learned behaviors^17,21–28^.

Scaffold proteins in the Dlg family (PSD95/Dlg4, PSD93/Dlg2, SAP102/Dlg3, SAP97/Dlg1) have been shown to be essential for assembly of postsynaptic complexes^18,29^, however, the requirement of DLGAP1 for the organization of protein interactions at the PSD has not been directly examined. DLGAP1 links Dlg family scaffolds to SHANK (SH3 and multiple ankyrin repeat domains proteins) family proteins (SHANK1-3), and protein complexes from these three families of scaffold proteins have been shown to be enriched in a variety of psychiatric and other complex brain disorders^30^. Therefore, *Dlgap1* deletion could disrupt the organization of the PSD by uncoupling these families of scaffold proteins. Furthermore, DLGAP1 is most highly expressed within neocortex, hippocampus, olfactory bulb, and cerebellum^31,32^, brain regions which have relevance to symptoms observed in neuropsychiatric disorders.

We evaluated *Dlgap1* wild type (WT), heterozygous (HT), and knockout (KO) mice for protein interactions within the PSD, and behavioral phenotypes relevant to OCD, ASD, and/or schizophrenia. OCD is characterized by obsessions, compulsions, or both^33^; schizophrenia is characterized by positive symptoms, including hallucinations and delusions, and negative symptoms, including anhedonia and social deficits^34^; core features of ASD include repetitive behaviors, and social deficits^35^. We assessed mice for sociability, grooming behaviors, sensorimotor gating, exploratory behavior, anxiety, locomotion, nest building, anhedonia, and depression-like behavior.

## Methods

### Animals

*Dlgap1* KO mice on a mixed 129/C57BL/6 J background were obtained from Dr. Seth Grant at Edinburgh University. Mice were backcrossed to C57BL/6 J mice for one generation and then HT × HT breeding produced experimental cohorts. Adult male and female *Dlgap1* WT, HT, and KO mice were subjects. Mice were maintained on a 12-hour light/dark schedule with food and water available ad libitum, and all tests occurred during the animals’ light cycle. All methods were performed in accordance with the local Institutional Animal Care and Use Committee (IACUC) guidelines and regulations.

Several cohorts were used for studies. Cohort 1 consisted of mice (n = 16/sex/genotype) aged 8–11 weeks at the start of experiments. Mice were assessed in the following tests: open field, dig test, splash test (week 1); social approach, nest building (week 2); prepulse inhibition (PPI) (week 4); forced swim test (week 6). Cohort 2 consisted of mice (males: 14 WT, 14 HT, 12 KO; females: 14 WT, 15 HT, 13 KO) aged 8–11 weeks at the beginning of testing. Mice were tested for sociability and sucrose preference. Cohort 3 consisted of 6 KO and 6 WT male mice (2–4 months), and were used for biochemical studies of the PSD. Sample sizes were selected based on estimates from our previous work^36–38^.

### Biochemistry

Immunoprecipitation of the DLG layer of the PSD scaffold was performed using a pan-Membrane Associated Guanylate kinase family (MAGUKs) antibody and determining the levels of SHANK proteins by HPLC-MS/MS. PSD-enriched fractions were prepared using a 3-step protocol as described previously^16^. Briefly, adult mouse cortex was homogenized, homogenates were spun for 4 min at 500 g, the supernatant was collected and centrifuged at 10,000 g, and the membrane fraction was solubilized. Solubilized membranes were centrifuged at 30,0000 RPM in a Beckman Optima Max rotor MLA-130 for 40 minutes, pellet was collected and solubilized. Enrichment and quality of PSD fractions was monitored against a number of protein controls including glutamate receptor subunits, presynaptic markers, cytoplasmic proteins, and core PSD scaffolds as previously described^16^. Samples were separated by NuPAGE® Novex® 4–12% Bis-Tris Gels and subsequently fixed and then stained with Instant blue®. Lanes were cut and placed into 96-well plates for de-staining, and digested by tripsin at 37 °C for 1 hour. Peptides were then extracted with acetonitrile. Peptide desalting and reverse phase separation of peptides was performed using the Nano/Capillary Liquid Chromatography (LC) System Ultimate 3000 (Thermo/Dionex) and samples then processed in a hybrid linear ion trap–Fourier transform ion cyclotron resonance (FTICR) mass spectrometer (MS). Antibodies for immunoisolation were screened for specificity using *Dlgap1* KO and *Shank3c*-terminal deleted KO mice, and anti-GST as negative controls^17^. Antibody concentrations were standardized for optimum protein recovery within ranges: 0.4–0.8 ug/ul. Immunoprecipitations were performed as described previously^16,30^, with minor modifications. Protein interactions were considered positive if at least two unique peptides were present in duplicate assays and absent in controls. MS data were processed using Proteome Discoverer 1.4 (PD, Thermo Scientific) and searched by both Sequest and Mascot V2.4 (Matrix science) against a modified mouse database that was downloaded from Uniprot and was combined with its decoy database. The mass tolerance used for searching was set as 10 ppm for precursor ions and 0.8 Da for fragment ions. No more than two missed cleavage sites were allowed. Static modification was set as cysteine carboxyamidation and dynamic modification was set as methionine oxidation. False discovery rates (FDR) were automatically calculated by the Percolator node of PD based on decoy database hits. A peptide FDR of 0.01 were used for cut-offs. Peptides with high confidence were considered as true hits and proteins with at least two different peptides were accepted.

### Open Field Test

Mice were placed into the corner of automated activity chambers, which were 41 cm × 41 cm with a 16 × 16 photobeam grid, and 2.54 cm between each photobeam^39^ (Accusan, Columbus, OH). Activity was recorded for 60 minutes. Total distance traveled was quantified to assess locomotor activity. Vertical activity was used to assess rearing, an exploratory behavior. Percent center distance was calculated as [(center distance/total distance) × 100] and was used as a measure of anxiety, with increases in center distance interpreted as a decrease in anxiety. Spatial *d* was calculated using BMDP, Python, and Night Owl to assess the degree to which consecutive movements were along a straight line (*d* ≈ 1), meandering (*d* ≈ 1.5), or contained directional changes (*d* ≈ 2)^40^.

### Dig Test

Immediately following the open field test, mice were placed into a novel cage with 1″ of standard, clean bedding and behavior was videotaped for 3 minutes. Videos were then scored by an experimenter blind to genotype and sex. Measures were: latency to dig, total time digging, number of bouts digging, and average bout duration. A digging bout was defined as significant displacement of bedding due to limb or nose movement lasting at least one second, and not separated by more than one second of rest. Increases in digging behavior indicate increased exploration^41^.

### Splash Test

Following the dig test, mice were allowed to habituate to the test cage for an hour. Mice were then removed from the test cage, sprayed twice on their dorsal surface from approximately 5″ away with a 10% sucrose solution, and returned to the test cage^42^. Behavior was videotaped for 5 minutes, and then scored by a blind experimenter for latency to groom, total time grooming, number of grooming bouts, and average bout duration. A grooming bout was defined as any number of leg strokes along the body, a minimum of 2 arm strokes over the face/head, or any amount of time spent licking/biting the fur, with a break not separated by more than 2 seconds. Decreases in grooming in response to sucrose spray was interpreted as increased anhedonia^43^.

### Nest Building

Nest building was used to assess general home cage behavior. Mice were placed into a novel cage with an unused nestlet (a 2″ × 2″ square of cotton fiber; Ancare). Nests were photographed at 0, 2, 4, and 6 hrs. Nests were scored by an experimenter blinded to genotype and sex, as described previously^44^. Briefly, nests were given a score of 0–5 according to the following criteria: 0 = untouched; 1 = minimal pieces ripped off; 2 = major pieces of nestlet square left intact; 3 = all/most ripped up but not organized; 4 = all ripped up, organized into clear nest but not perfect, no tight walls; 5 = all ripped up, organized into clear nest, tight walls^45^. Additionally, nests were weighed at time 0 and 6 hours, and the amount of nestlet used was calculated by subtracting the intact portion of the nestlet remaining at 6 hours from the original nestlet weight at time 0.

### Prepulse Inhibition

Prepulse inhibition was used as an index of sensorimotor gating, which is deficient in patients with schizophrenia^46,47^, ASD^48,49^, and OCD^50,51^. Mice were placed into startle chambers that consisted of a Plexiglas cylinder resting on a Plexiglas platform in a sound-attenuating, ventilated chamber (San Diego Instruments, CA). Sessions were 20 minutes long. The first 5 minutes consisted of acclimation to the background (65 dB) noise. The testing consisted of a pseudo random representation of five different types of trials: a 40-millisecond broadband 120 dB burst (pulse alone trial); three different prepulse pulse trials in which 20-millisecond long 3 dB, 6 dB, or 12 dB above background stimuli preceded the 120 dB pulse by 100 milliseconds (onset to onset); and a no stimulus trial, in which only background noise (65 dB) was presented^52,53^. PPI was calculated as [(startle amplitude_pulse_ − startle amplitude_prepulse + pulse_)/startle amplitude_pulse_) × 100].

### Forced Swim Test

The forced swim test was used as an index of a depression-like behavior. Mice were placed into a bucket (24 cm high and 19 cm in diameter filled 19 cm high) filled with 23 °C to 25 °C tap water. Behavior was videotaped from above for 6 minutes, with the last four minutes scored by a blinded experimenter for time climbing, swimming, and immobile. Mice were also exposed to a 6-minute pretest 24 hours earlier. An increase in immobility or decrease in active behavior reflects a depression-like phenotype^54^.

### Social Approach Experiment 1

Cohort 1 mice were habituated to open field chambers (106 × 106 × 76 cm; Accuscan, Columbus, OH) for 5 minutes. The test mouse was then removed, and a novel mouse (C57BL/6 J mouse of same sex, but lower body weight) was placed in a clear plastic cage (10 L × 8 W × 12 H cm) in one corner of the chamber. A novel object [a clear plastic cage (10 L × 8 W × 12 H cm)] was placed in the opposite corner of the chamber. The test mouse was then placed back in the chamber for an additional 5 minutes and allowed to move freely throughout the chamber. Data was collected automatically within the open field and specifically within the interaction area, defined as a 5 cm radius around the novel object or novel mouse. Dependent measures were entries, vertical time, total distance traveled, and time spent within the interaction area.

### Social Approach Experiment 2

Cohort 2 mice were run in another social approach paradigm to assess number of sniffs made towards the novel mouse versus novel object. Mice were allowed to habituate to open field chambers for 10 minutes. During habituation, two wire cups (H: 10.5 cm, D: 9.5 cm) were present, centered along the left and right wall of the chamber. Following the 10-min habituation period, the test mouse was removed. A novel object (lego) was placed under one cup and a novel mouse was placed under the other cup. The novel mouse was a C57BL/6 J mouse of the same sex, but lower weight than the test mouse. The position of the mouse and object were counterbalanced among test mice. The test mouse was then returned to the open field chamber for 10 minutes. Behavior was videotaped and scored blindly for latency to first sniff, and number of sniffs. The chamber, cups, and novel object were cleaned between each mouse with 70% isopropyl alcohol. Since latency data was not normally distributed, latency data were log_10_ transformed.

### Sucrose Preference

Mice were individually housed into assigned test cages, where they had access to water and sucrose in separate bottles for 4 hours. The concentrations of sucrose (0.5, 1, and 2%) were tested on sequential days, in ascending order, with the left/right sucrose bottle side switched each session. Volumes were recorded before and after each session, and sucrose preference was calculated as [(ml sucrose/ml sucrose + water) * 100].

### Statistical Analyses

Two-way ANOVAs were performed for all measures with sex and genotype as factors. Main effects of genotype were resolved using Student Newman-Keuls post hoc tests. Interactions were resolved using one-way ANOVAs to compare the sexes, and Student Newman-Keuls post hoc tests to compare genotypes. For all measures, outliers were removed if their value was greater than two standard deviations above or below the mean. Alpha was set at 0.05. Variance was comparable between all compared groups.

## Results

### Biochemistry

**Figure 1:**
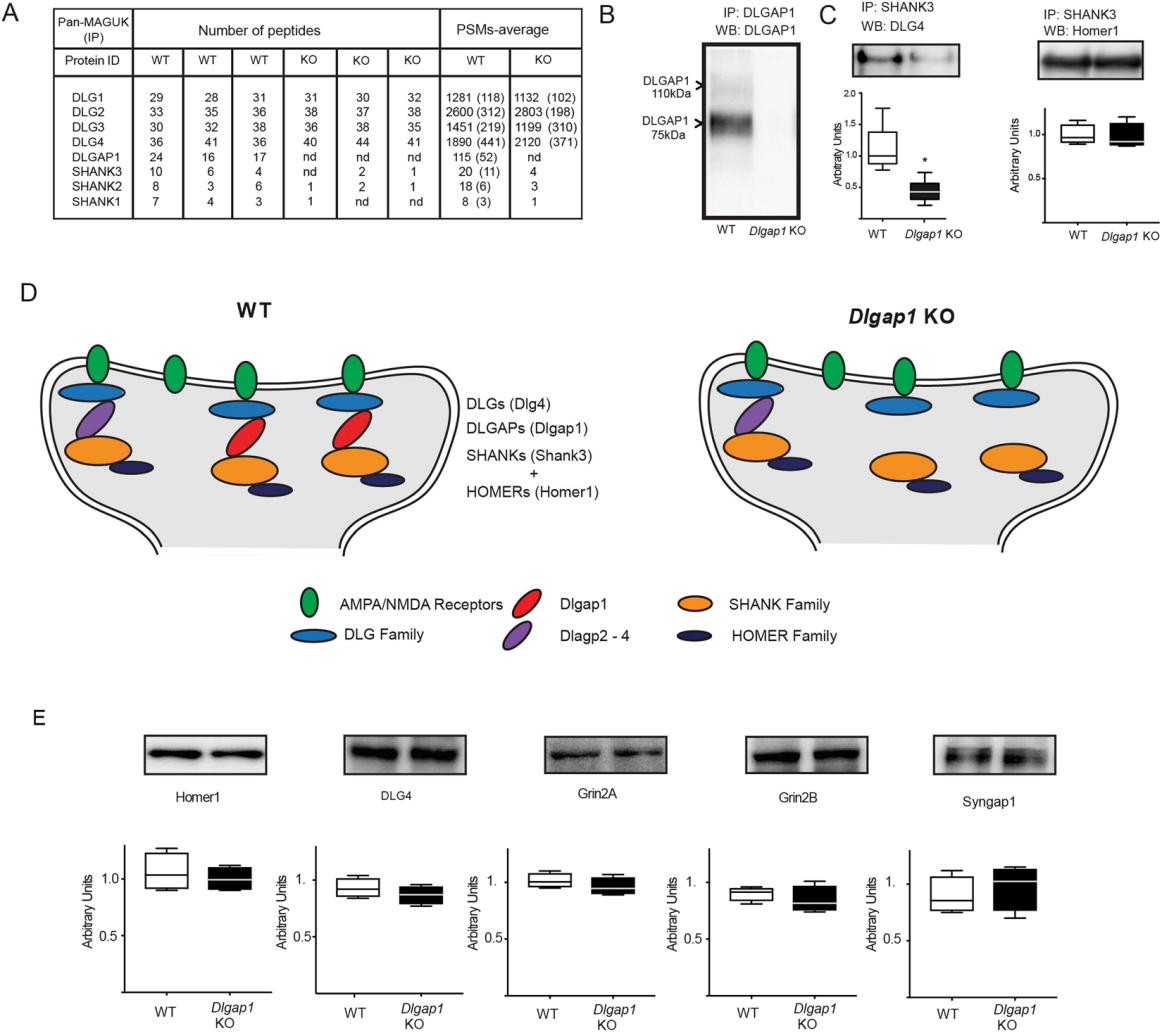
(**A**) The number of unique peptides identified in pan-MAGUK immunoprecipitation (IP) assays detected by HPLC-MS/MS from adult mouse cortex are shown. Columns show results from triplicate experiments, protein ID (Gene name), number of unique peptides and number of peptide spectrum matches (PSMs) (average of triplicate assays) for WT and *Dlgap1* KO mice. (**B**) Representative blots showing two major isoforms of DLGAP1 in WT and their absence in *Dlgap1* KO adult mouse cortex. (**C**) Representative blot of triplicate assays showing decreased (p = 0.0080, two-tailed non-parametric t-test) levels of DLG4 (left), and no changes in Homer1 in Shank3 IP in *Dlgap1* KO adult mouse cortex (right). (**D**) Cartoon shows scheme of three layers of PSD scaffold proteins. Top: DLGs (Dlg1-4), Middle: DLGAPs (DLGAPs1-4) and Bottom: SHANKs (Shank1-3). Cartoons indicate decreased association of top and bottom layers in *Dlgap1* KO (right) relative to WT (left). (**E**) Box plots and representative western blots of total protein levels of Homer1, Dlg4, Grin2A, Grin2B and Syngap1 in WT and *Dlgap1* KO adult mouse cortex. No changes in total protein levels were observed.

Within the PSD of excitatory glutamatergic synapses, a simplified scheme can describe a core scaffold structure (Fig. 1D), with three main groups of scaffold proteins: (1) an “upper layer” of scaffold proteins associated with glutamate receptor ion channels, including DLGs. (2) a “bottom layer” of scaffold components, including the SHANKs 1–3, and (3) an intermediate layer of scaffold molecules, functioning as a link between the “upper and lower layers”. This “middle layer” includes the disk large associated protein family (DLGAP1-4)^17^.

As a first approach, we asked if the deletion of *Dlgap1* impairs the interaction between the three scaffold-layers of the PSD. We immunoprecipitated DLG scaffolds with a pan-MAGUK antibody^30^ and identified their protein interactions by HPLC-MS/MS in WT and *Dlgap1* KO mouse cortex. Figure 1A shows the number of unique peptides, run in triplicate, identified in pan-MAGUK immunoprecipitation (IP) as well as number of peptide spectrum matches (PSMs) (average of triplicate assays). Figure 1B shows that a pan-MAGUK antibody was able to coimmunoprecipitate DLGAP1 and SHANKs (SHANK1,2,3) together with the DLG family of scaffolds (DLG1,2,3,4) in WT cortex. This finding is consistent with previous results indicating that DLG proteins directly bind DLGAP1^55^, which in turn binds to SHANK proteins^56^. Moreover, our previous studies show that DLG4, DLGAP1 and SHANK3 form large protein interaction complexes with each other^30^. Here, we also found that immunoisolation of DLG scaffolds from *Dlgap1* KO mice recovered few SHANK1-3 peptides, which were below the level of detection (Fig. 1A).

To confirm that DLGAP1 was required for connecting DLG and SHANK proteins in PSD supercomplexes, we immunoprecipitated SHANK3 from WT and *Dlgap1* KO mice cortex and performed Western Blots for Dlg4 (Psd95). Figure 1B,C shows that *Dlgap1* deletion impairs 59.6% (p = 0.0080) of the association of Dlg4 to Shank3 supercomplexes. Since Shank3 complexes also contain other members of the DLGAP family in adult mouse cortex such as DLGAP2, DLGAP3, and DLGAP4^30^, a fraction of Shank3 protein can still associate to DLG supercomplexes through interaction with DLG2, 3 and 4 (Fig. 1D). Moreover, while SHANK3 connection to DLG upper scaffolds was impaired in *Dlgap1* KO, the interaction of SHANK3 with the downstream component Homer1 was unaffected (p = 0.823) (Fig. 1E), indicating that DLGAP1 selectively associates DLGs to SHANKs supercomplexes.

Finally, we asked if changes in protein interactions in *Dlgap1* KO mice were a consequence of general changes in total protein levels. Figure 1E shows that NMDAR receptors subunits, Grin2A and Grin2B and PSD scaffolds and adaptors DLG4, SynGAP1, and Homer1 were unchanged in *Dlgap1* KO mice versus WT mice.

### Open Field Test

**Figure 2:**
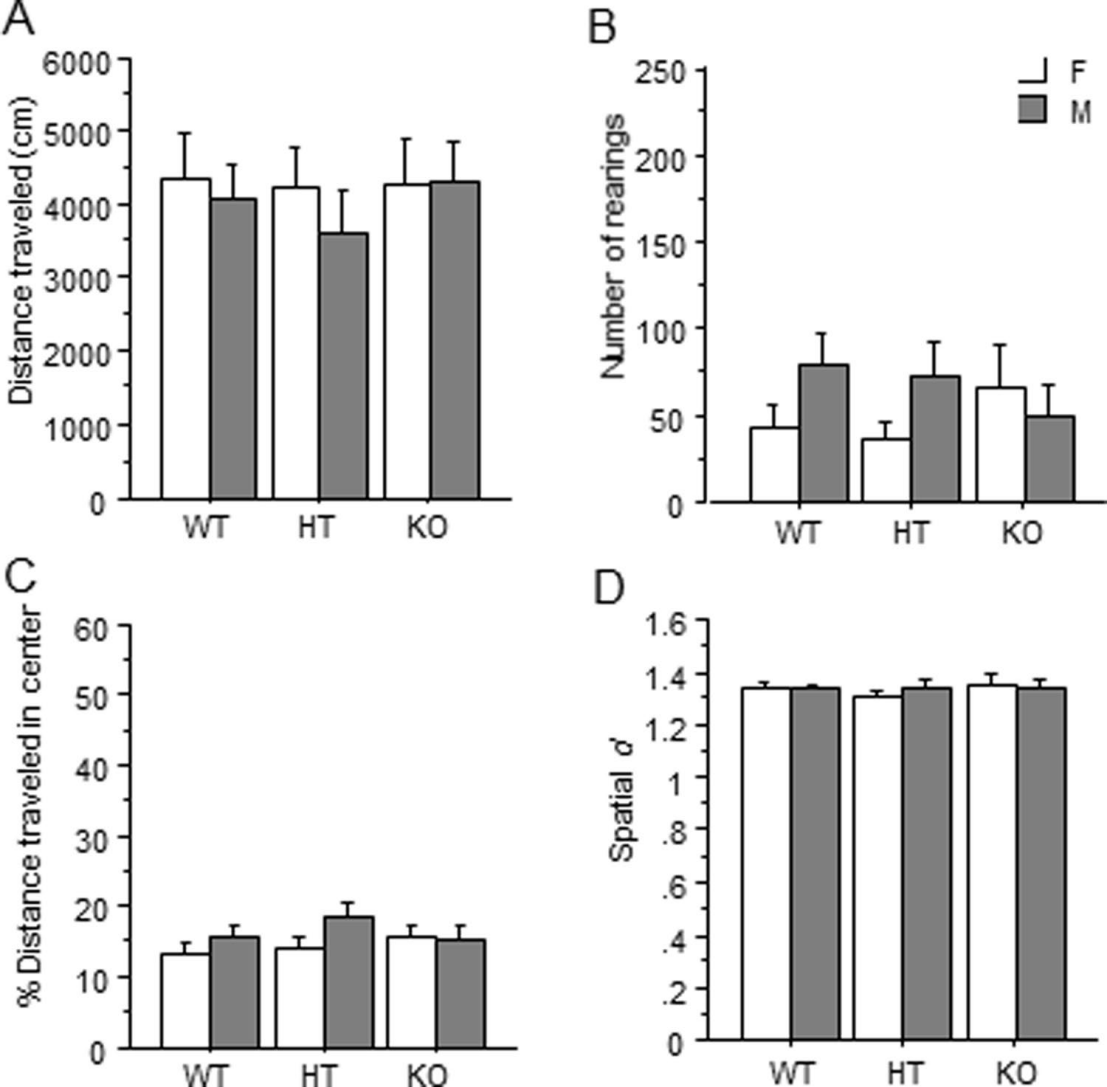
No effect of *Dlgap1* genotype in the open field test for (**A**) total horizontal distance, (**B**) vertical activity, (**C**) percent center distance, or (**D**) spatial *d* score.

The measures of total distance traveled (Fig. 2A), rearing (Fig. 2B), percent of total distance traveled in the center (Fig. 2C), and spatial *d* (Fig. 2D) were not altered by genotype or sex. No interactions were observed.

### Dig Test

**Figure 3:**
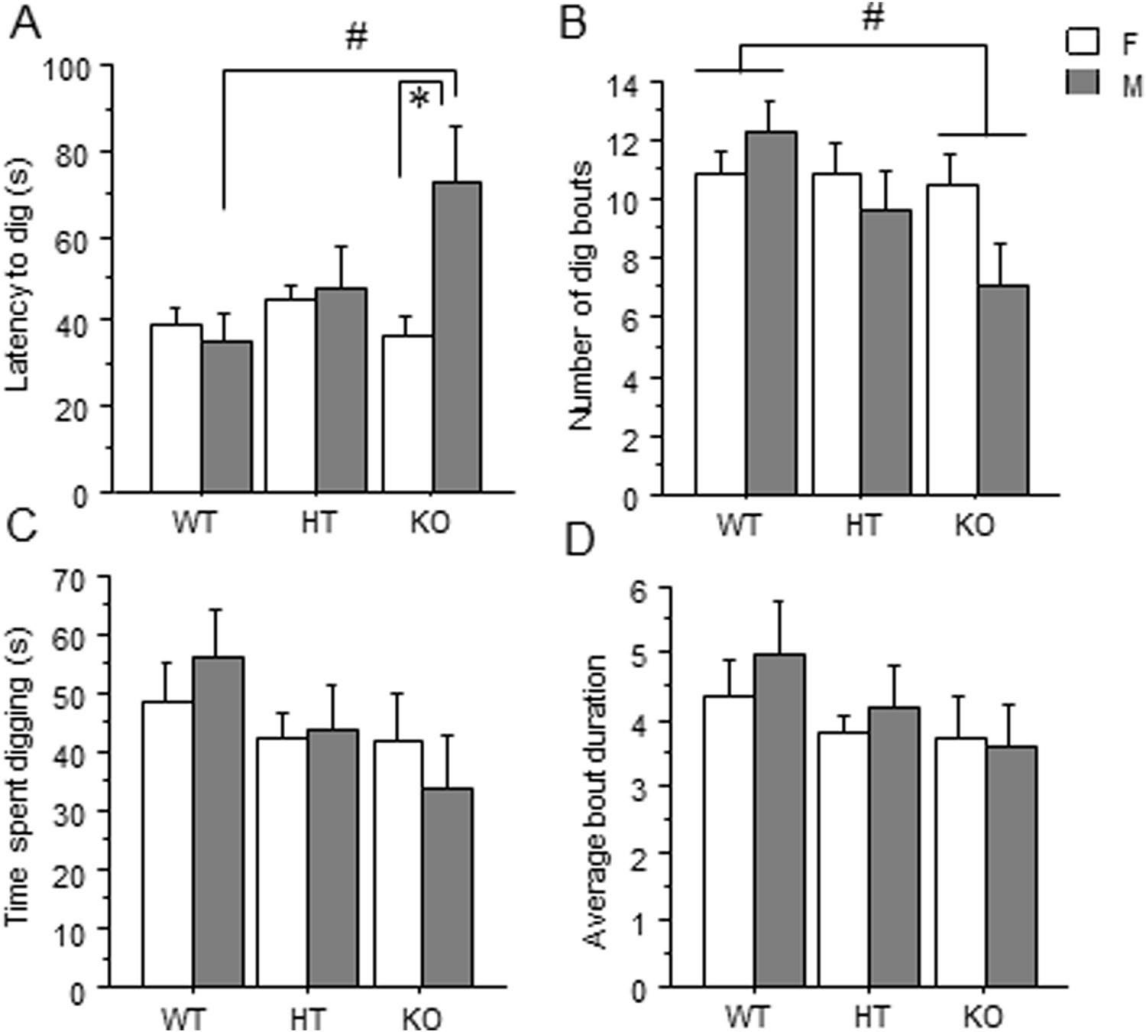
Effect of *Dlgap1* genotype in the dig test on (**A**) latency to dig, (**B**) number of dig bouts, (**C**) total time spent digging, and (**D**) average digging bout duration. *p < 0.05.

A genotype by sex interaction [F(2,88) = 3.485] (*p* < 0.05) (Fig. 3A) and post hoc tests revealed an increased latency to dig in male *Dlgap1* KO mice compared to male WT mice [F(2,45) = 3.31] (p < 0.05). Furthermore, male *Dlgap1* KO mice had longer latencies to dig than female KO mice (p < 0.05). A main effect of genotype on number of digging bouts was also found [F(2,90) = 3.12] (p < 0.05) (Fig. 3B), with *Dlgap1* KO mice exhibiting fewer digging bouts than WT mice (p < 0.05). Neither total time spent digging (Fig. 3C) nor average bout duration (Fig. 3D) were affected by genotype or sex.

### Splash Test

**Figure 4:**
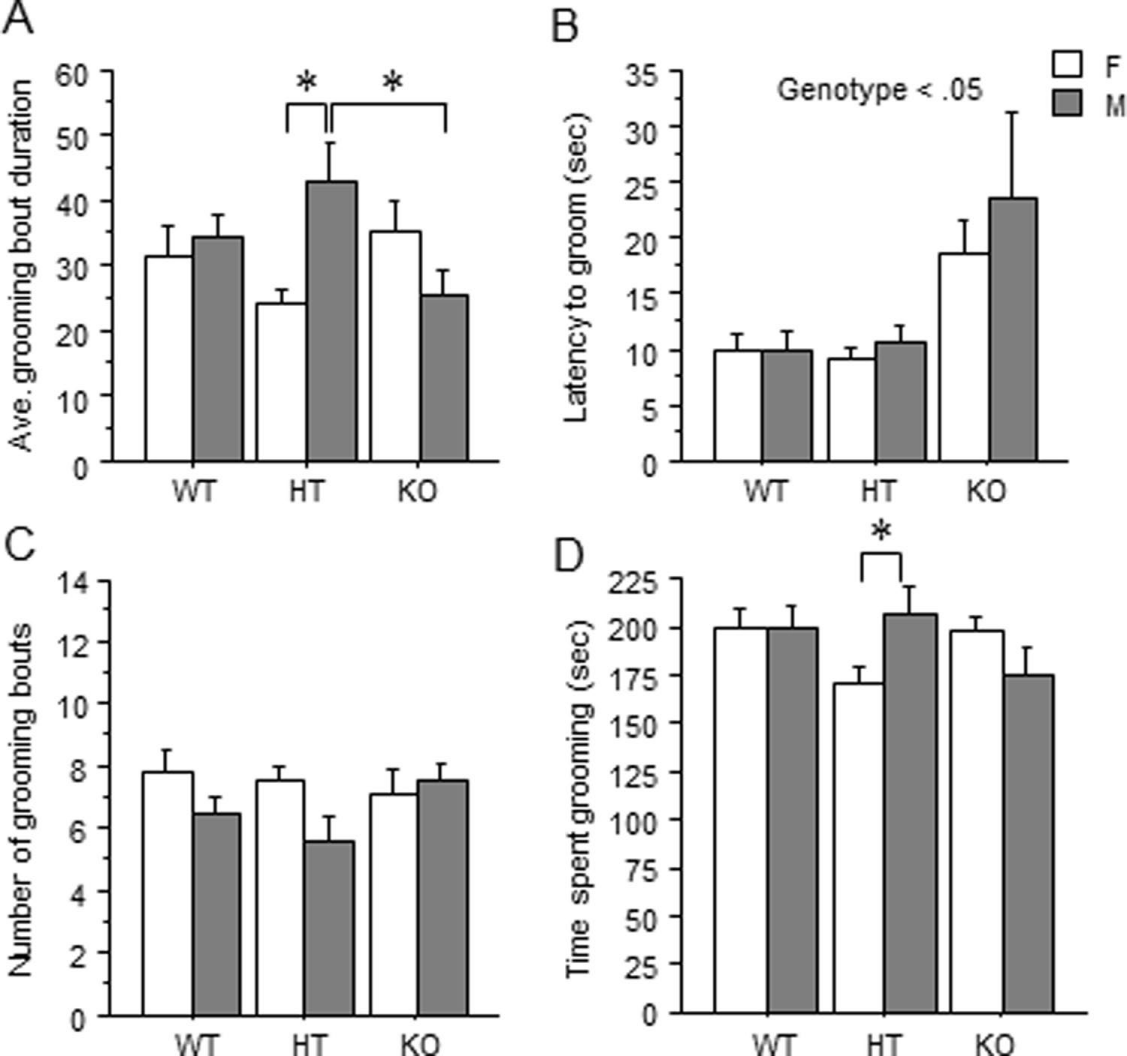
Effect of *Dlgap1* genotype in the splash test on (**A**) average bout duration (**B**) latency to groom (**C**) number of grooming bouts, and (**D**) total time spent grooming. *p < 0.05.

Analysis revealed a genotype by sex interaction for average bout duration [F(2,88) = 5.70](p < 0.01) (Fig. 4A). Post hoc tests showed that within males, *Dlgap1* KOs had fewer grooming bouts than HTs [F(2,43) = 4.02] (p < 0.05). Additionally, within HTs, males had longer grooming bouts than females (p < 0.05). Furthermore, *Dlgap1* KO mice showed a longer latency to groom compared to either WT or HT mice [F(2,88) = 6.25] (*p* < 0.01) (Fig. 4B). No main effects or interaction of sex and genotype were observed for the number of grooming bouts (Fig. 4C). A genotype by sex interaction and post hoc tests revealed that within *Dlgap1* HTs, males spent more time grooming than females [F(2,88) = 3.60] (p < 0.05) (Fig. 4D).

### Nest Building

Across groups, nest quality increased over time (p < 0.001) (Supp. Figure 1A). The average nest quality score was rated at 2.6 at the 6-hour time-point. Therefore, either increases or decreases in nest quality should have been detectable in *Dlgap1* KO mice compared to WT mice on the 5 point scale used, with 2.6 representing an intermediate score. However, no main effects or interaction of sex and genotype were found for nest quality. Similarly, the amount of nestlet used by the 6-hour time-point did not differ between groups (Supp. Figure 1B).

### PPI

Neither genotype nor sex affected baseline startle (data not shown) or PPI (Supp. Figure 2). There were no interactions including genotype, sex, or prepulse intensity.

### FST

No interactions or main effects of genotype or sex were found for time immobile (Supp. Figure 3A), climbing (Supp. Figure 3B), or swimming (Supp. Figure 3C). There was a main effect of sex on climbing, in which males climbed more than the females [F(2,90) = 7.17] (p < 0.01) (Supp. Figure 3B).

### Social Approach

**Figure 5:**
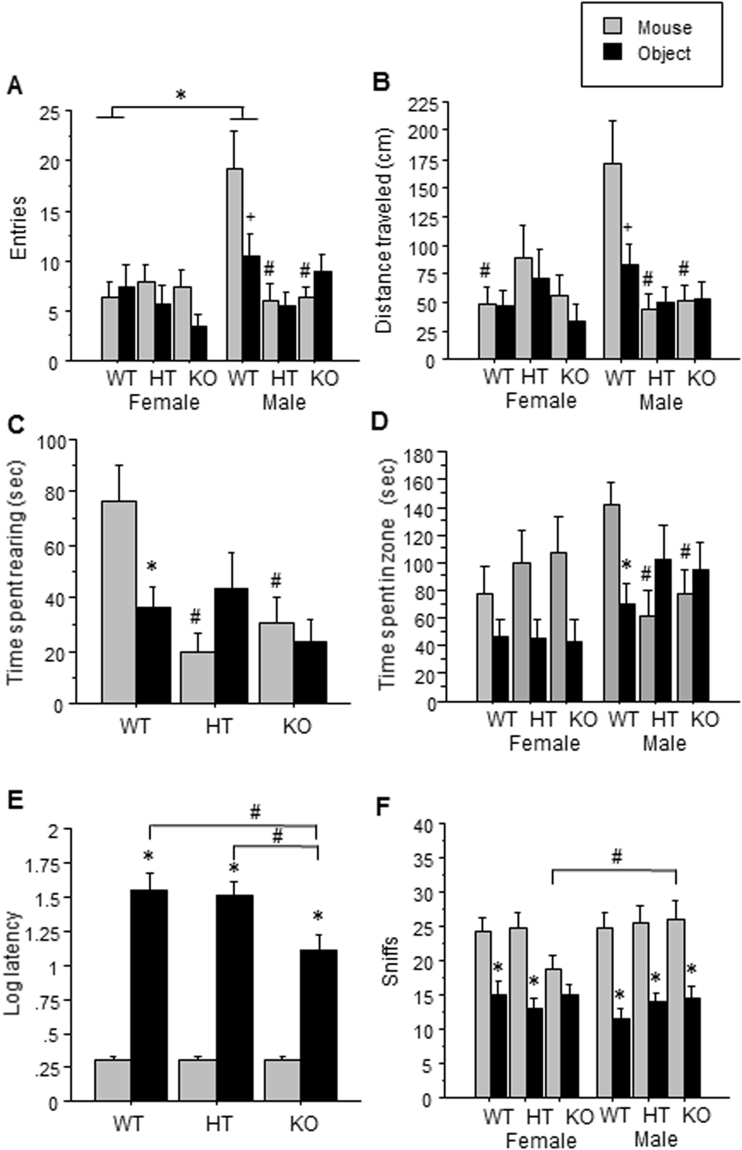
Effect of *Dlgap1* genotype on social approach in the first social interaction test for (**A**) number of entries, (**B**) total distance traveled, and (**C**) total time spent rearing, and (**D**) total horizontal time, all with respect to the zone containing the novel mouse or the object. Effect of *Dlgap1* genotype in the second social interaction test for (**E**) log latency to the first sniff and (**F**) number of sniffs with respect to the mouse or the object. *Indicates a significant difference between conditions (mouse vs. object) (p < 0.05); +p indicates a difference between conditions at the trend level (0.05 < p < 0.10); ^#^indicates a significant difference between genotype or sex (p < 0.05).

Experiment 1: For the number of entries, a three-way interaction of sex by genotype by condition (mouse vs. object) [F(2,90) = 4.83] (p < 0.01) (Fig. 5A) and post hoc tests showed that within males, both *Dlgap1* HTs and KOs made fewer entries into the zone containing the mouse than WTs, indicating less sociability (p < 0.05). In addition, male WTs made more entries into either zone (containing the mouse or object) than female WTs [F(2,90) = 4.81] (p < 0.01), indicating increased general exploration. The greater number of entries made into the zone containing the mouse versus the object by male WT mice did not achieve statistical significance [F(1,15) = 3.56] (p = 0.08). For distance traveled, a three-way interaction of sex by genotype by condition [F(2,90) = 3.45] (p < 0.05) (Fig. 5B) and post hoc tests indicated that male *Dlgap1* WTs traveled more distance near the mouse than female WTs [F(2,45) = 3.68] (p < 0.05), indicating more social approach. Furthermore, male HT and KO mice showed reduced distance traveled in the zone containing the mouse than male WT mice, indicating reduced social approach. The increased distance traveled around the mouse versus the object by male WT mice did not achieve statistical significance [F(1,15) = 4.33] (p = 0.05). For time spent rearing, an interaction of genotype by condition and post hoc analysis showed that WT mice spent more time rearing near the mouse than near the object, while *Dlgap1* HT and KO mice did not [F(2,90) = 6.12] (p < 0.01) (Fig. 5C, Supp. Figure 4). Furthermore, WT mice spent more time rearing around the mouse than HT or KO mice. Finally, there was an interaction of sex by condition [F(1,90) = 4.22] (p < 0.05). Post hoc tests showed that female, but not male, mice spent more time rearing around the mouse than the object [F(1,47) = 6.93] (p < 0.01), and females spent less time rearing around the object than males (Supp. Figure 5) (P < 0.05). For horizontal time in the interaction zone, a trend for interaction of genotype by sex by condition was found [F(2,88) = 2.81] (p = 0.07). Post hoc tests showed that across genotypes, females mice spent more time in the zone surrounding the mouse versus the object [F(1,88) = 10.15] (p < 0.01) (Fig. 5D). Within males, WTs spent more time in the zone around the mouse than the object [F(2,88) = 4.59] (p < 0.05). Lastly, Newman Keuls post hoc tests showed that male WT mice spent more time around the mouse than male HT or KO mice. For the measure of duration, one male WT and one female WT were outliers, and were excluded from analysis.

Experiment 2: For the log latency to the first sniff, a genotype by condition interaction [F(2,73) = 4.83] (p < 0.01) (Fig. 5D; Supp. Figure 6) and post hoc tests revealed that each genotype showed a shorter latency to sniff the mouse than the object (all p’s < 0.0001); however, *Dlgap1* KO mice showed a shorter latency to sniff the object than WT and HT mice (p < 0.05). For the number of sniffs, no main effects or interactions were found. However, planned comparisons revealed that within each genotype and sex, all groups sniffed the mouse more than the object (all p’s < 0.008), with the exception of female *Dlgap1* KO mice (Fig. 5E). Additionally, *Dlgap1* KO females sniffed the mouse less than *Dlgap1* KO males (p < 0.05).

### Sucrose Preference

Sucrose preference was not altered by genotype or sex, and no interactions were found (Supp. Figure 7).

### Data availability

The datasets generated during and/or analyzed during the current study are available from the corresponding author on reasonable request.

## Discussion

Here we show that *Dlgap1* KO mice exhibit impairments in the physical organization of the PSD and selective deficits in sociability. Alterations of the PSD in *Dlgap1* KO mice included loss of association between core scaffold proteins that are components of postsynaptic signaling complexes. We also observed deficits in sociability in *Dlgap1* KO mice using two distinct behavioral paradigms that rely on different dependent measures. One paradigm used automated data collection and measured horizontal time, number of entries, distance traveled, and vertical time within a zone proximal to the target mouse or object; the other paradigm assessed sniffing of the target mouse or object rather than mere proximity. *Dlgap1* KO male mice also showed reductions in exploratory behavior in the dig test, and increased anhedonia in the splash test. However, these findings were not corroborated by other tests assessing similar constructs. For example, no differences in exploration were found in the open field test, and no differences in anhedonia were found in the sucrose preference test. Furthermore, no effect of genotype was found for locomotor behavior, nest building, startle reactivity, forced swim test behavior, or prepulse inhibition. Therefore, disorganization of specific protein complexes within the PSD and social approach deficits constitute selective deficits observed in *Dlgap1* KO mice. Our findings are consistent with reports suggesting that genetic variation decreasing the function of *DLGAP1* might contribute to risk for both schizophrenia^13^ and ASD^9^, disorders characterized by social deficits^57,58^.

Our biochemical results reveal that DLGAP1 is involved in connecting the top and bottom layers of the PSD scaffold machinery (Fig. 1). Immunoprecipitation of SHANK3 showed a decrease in the ternary association to DLG4 through DLGAP1. Moreover, immunoisolation of DLG scaffolds recovered few peptides for SHANK1-3 which could not be detected and quantitated by HPLC-MS/MS in all the replicates of *Dlgap1* KO mice (Fig. 1A). These findings suggest a general impairment in the association of SHANKs and DLGs supercomplexes in *Dlgap1* KO mice. Furthermore, we observed similar levels of the NMDAR receptors subunits Grin2A and Grin2B, PSD scaffolds, and adaptors DLG4, SynGAP1, and Homer1 between *Dlgap1* WT and KO mice, suggesting that the alteration in protein interactions between the top and bottom layers of the PSD scaffold in *Dlgap1* KO mice is not due to alterations in the levels of these proteins at the PSD. These protein interactions are influenced by several factors, including proteins’ binding affinities and the local concentration of proteins. One possibility is that lack of DLGAP1 frees available binding sites for other members of the DLGAP family (DLGAP2-4) to interact with DLG and SHANK family members, producing “novel” and perhaps abnormal PSD interactions. Thus, changes in the copy number of scaffolds such as DLGAP1 might increase or decrease the total number of “slots” available for interactions, and strongly influence PSD signaling machinery^59^. Furthermore, the interaction between SHANK3 and Homer1 was found to be unaltered in *Dlgap1* KO mice. Homer1 has recently been implicated in depression-related behaviors in rodents^60,61^ and humans^62^. Our results indicating normal interactions between SHANK3 and Homer1 in *Dlgap1* KO mice is consistent with the absence of any depression-related phenotype in these mice, including lack of effects in the FST and sucrose preference test.

Animal models have previously implicated NMDA receptor hypofunction in deficits in sociability. For example, mouse models of NMDAR hypofunction, including knockdown of the NR1 subunit^63^ or tissue specific and/or inducible knockout of NR1^63–66^, have all reported reduced sociability. Specifically, knockout of NR1 in the forebrain during adulthood reduced social motivation^64^. Other genetic manipulations impairing NMDA receptor function, such as reduced NMDA glycine site affinity, also produce social deficits in mice^67^. Thus, the reduction in social approach observed in *Dlgap1 KO* mice might result from impaired NMDA receptor signaling due to disrupted organization of the supercomplexes containing NMDARs, DLG, DLGAP1 and SHANKs. In addition to the components of the supercomplexes, downstream effector proteins including AMPA receptors also play a role in social interactions. Indeed, AMPA receptors also regulate sociability^68,69^. Constitutive GluR1 receptor knockout mice show reductions in sociability^70^; however, deletion of GluR1 during late adolescence using a tamoxifen-inducible system leads to cognitive impairments, PPI deficits, and hyperlocomotion, but not social approach deficits^71^. Furthermore, haploinsufficiency of the ASD/SCZ associated gene *Shank3* leads to reduced sociability in mice^72^. Our results extend this finding by showing that the reduced social behavior *Dlgap1* KO mice also correlates with a deficit in SHANK3 protein interactions at the PSD (Fig. 1A). Thus, disruption of core scaffold proteins can contribute in a reciprocal manner to the disorganization of the core signaling machinery of the PSD and result in endophenotypes for psychiatric disorders.

We observed sex differences in the effect of *Dlgap1* genotype on sociability. For example, male, but not female, *Dlgap1* HT and KO mice showed reductions in horizontal time, vertical time, number of entries, and distance traveled in the region surrounding the target mouse (Fig. 5A,B). However, this finding is due in part to the greater horizontal time, number of entries, and distance traveled by WT males than WT females; thus, the lack of effect in females might be due to a floor effect (Fig. 5). However, female *Dlgap1* HT and KO mice showed reductions in vertical time spent in the region surrounding the target mouse compared to WT mice (Fig. 5C; Supp. Figure 4), indicating reduced social approach. Interestingly, in the second social interaction test, all *Dlgap1* genotypes showed a similar latency to sniff the mouse, but *Dlgap1* KO mice had a shorter latency to sniff the object than *Dlgap1* WT or HT mice, suggesting increased exploration of the object (Fig. 5E). Finally, female *Dlgap1* KO mice were the only group that did not sniff the mouse more times than the object (Fig. 5E). Thus, decreased *Dlgap1* expression reduced social approach in both sexes, although different measures were affected in males versus females. These findings demonstrate the importance of assessing multiple measures of sociability, since assessing only one could lead to an erroneous conclusion regarding sex differences in sociability. Future studies should further investigate the deficits in sociability in *Dlgap1* KO mice; for example, assessing ultrasonic vocalizations could determine whether this phenotype has an early onset^73^.

*Dlgap1* KO mice also showed alterations in several other behavioral measures. They showed decreased exploration in the dig test (Fig. 3); however, we did not find alterations in other exploratory behaviors, such as rearing in the open field test. We also assessed sensorimotor gating (PPI), which is reduced in patients with OCD, ASD, or schizophrenia, but did not observe any differences due to genotype in mice (Supp. Figure 2). Even though we found consistent reductions in the social approach of *Dlgap1* KO mice, we found few genotypic differences in other tests of anhedonia, such as the sucrose preference test. In the splash test, *Dlgap1* KO male mice showed evidence of anhedonia in only one measure, the latency to groom (Fig. 3F). Finally, *Dlgap1* KO mice show normal levels of locomotor activity (Fig. 2A), unlike the mice harboring null alleles of other PSD proteins such as SHANK2^74^ or SHANK3^75^. Since the consequences of gene knockout can vary on different genetic backgrounds^76^, more studies will be required to determine whether knockout of *Dlgap1* primarily affects social approach behavior in different mouse strains. Although *Dlgap1* HT and KO mice showed reductions in sociability, but not other behavioral abnormalities observed in schizophrenia or autism patients such as PPI deficits^46–49^, reduced *DLGAP1* function may still contribute to risk for these disorders. Recent human genetic studies indicate that the risk for neuropsychiatric disorders is sometimes conferred by single gene variants, but is often conferred by variation in many genetic loci, such that one gene contributes only to a portion of the behavioral syndrome^77,78^. Here, we suggest that reduction of *DLGAP1* function may contribute to the reduced sociability observed in disorders linked to this gene, including autism^9^ and schizophrenia^8,13–15^.

The *Dlgap* family has distinct regional expression patterns in the brain^31^. While *Dlgap1* is expressed at a high density in the cortex, a brain region implicated in social cognition^79^, *Dlgap3* is enriched in striatum^80^. Interestingly, *Dlgap3* has also been associated with OCD-related phenotypes^80^. Thus, behavioral phenotypes associated with mutations in *Dlgap* family members might better correlate to their relative expression levels in different brain regions, rather than to specific protein interactions within the PSD. Furthermore, other PSD elements, including the SHANK proteins^81^, are also differentially expressed across brain regions. Thus, although mutations in *Dlgaps* or *Shanks* might disrupt the scaffold structure of the PSD, the correlation between mutations and behavioral phenotypes might depend on the overlapping patterns of expression of each family member. For example, *Shank1* or *Dlgap1* mutations might correlate with behaviors dependent on cortical function, while *Shank3* or *Dlgap3* mutations may have a better correlation with behaviors modulated by striatal activity.

Our present findings show that lack of *Dlgap1* in mice disrupts protein interactions within the PSD of the cortex, and induces selective deficits in sociability. Our results suggest that one function of DLGAP1 protein is to interlink and connect scaffold proteins in the PSD. The reductions in sociability were highly specific, with no changes in locomotor activity, anxiety, sensorimotor gating, depression-like behaviors, or nest-building observed in *Dlgap1* KO mice. Yet, several reports suggest that alterations to PSD proteins can produce age-dependent changes in anxiety or depression-related behaviors^82,83^. Thus, our present results cannot rule out that DLGAP1 KO mice might have alterations in these phenotypes during the juvenile period, or following aging. Our findings are consistent with observations that individuals affected by schizophrenia or ASD carry rare genetic variants that are predicted to reduce the function of DLGAP1. In sum, the present findings extend upon previous reports that PSD dysfunction can lead to deficits in sociability. Future studies should further establish the precise mechanisms by which *Dlgap1* KO contributes to social deficits.

## Electronic supplementary material

Supplemental Figures
