# Supplementary material for: Dlgap1 knockout mice exhibit alterations of the postsynaptic density and selective reductions in sociability
Source: Sci Rep. 2018 Feb 2;8:2281. doi: 10.1038/s41598-018-20610-y (PMC5797244; doi:10.1038/s41598-018-20610-y)
Supplement: Supplementary file 1 — Supplemental Figures [file 41598_2018_20610_MOESM1_ESM.pdf]

Supplemental information

Dlgap1 knockout mice exhibit alterations of the postsynaptic density and selective reductions in sociability.

Coba MP[3], Ramaker MJ[1], Ho EV[1], Thompson SL[1,2], Komiyama NH[4], Grant SGN[4], Knowles JA[3], Dulawa SC[1]

Department of Psychiatry, University of California San Diego[1]

Committee on Neurobiology, The University of Chicago[2]

Department of Psychiatry and the Behavioral Sciences, Zilkha Neurogenetic Institute, Keck School of Medicine, University of Southern California[3]

Genes to Cognition Program, Centre for Clinical Brain Sciences, Edinburgh University[4]

Supplemental Figure 1

A) Nest Score

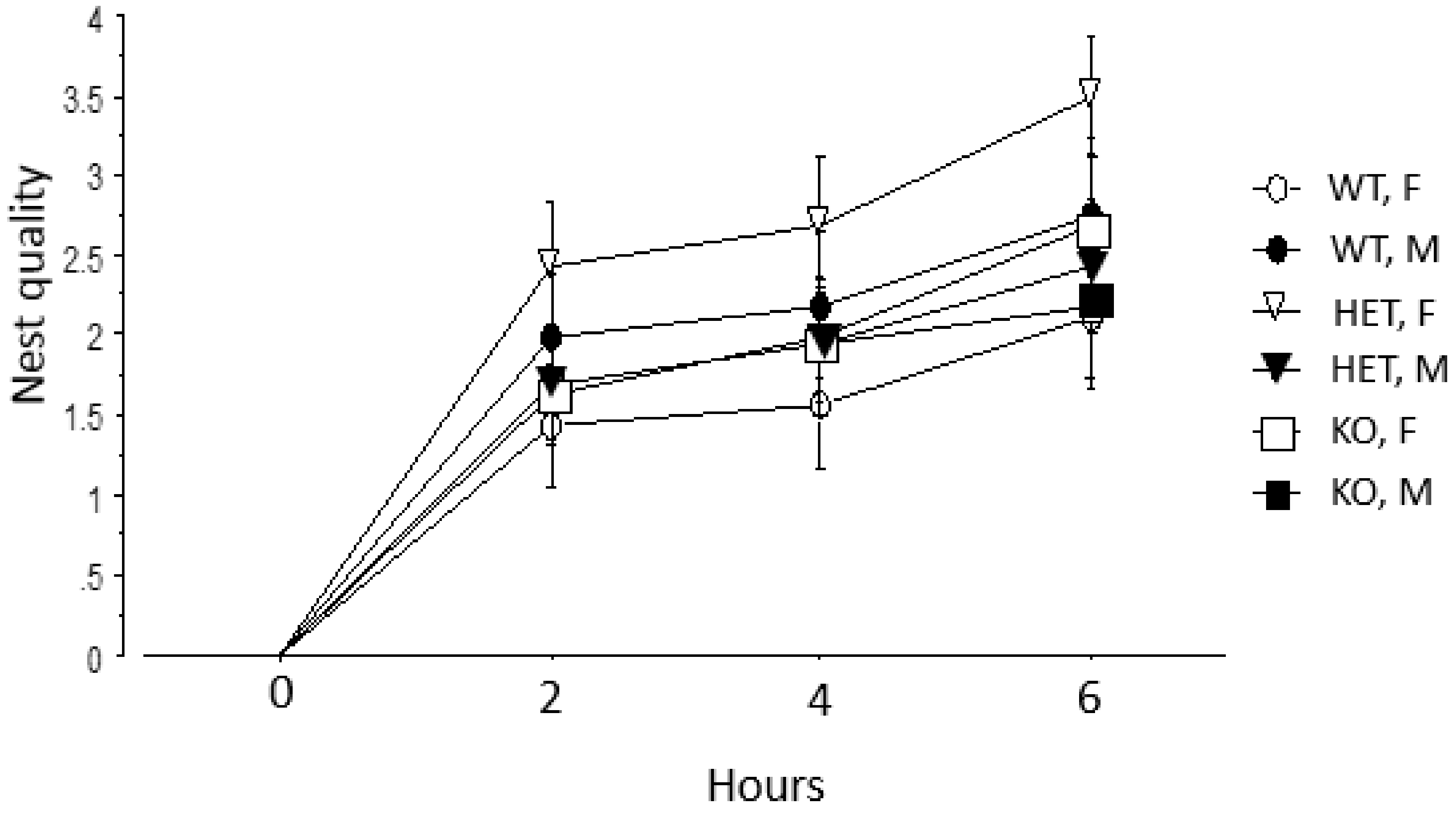

B) Nestlet Used

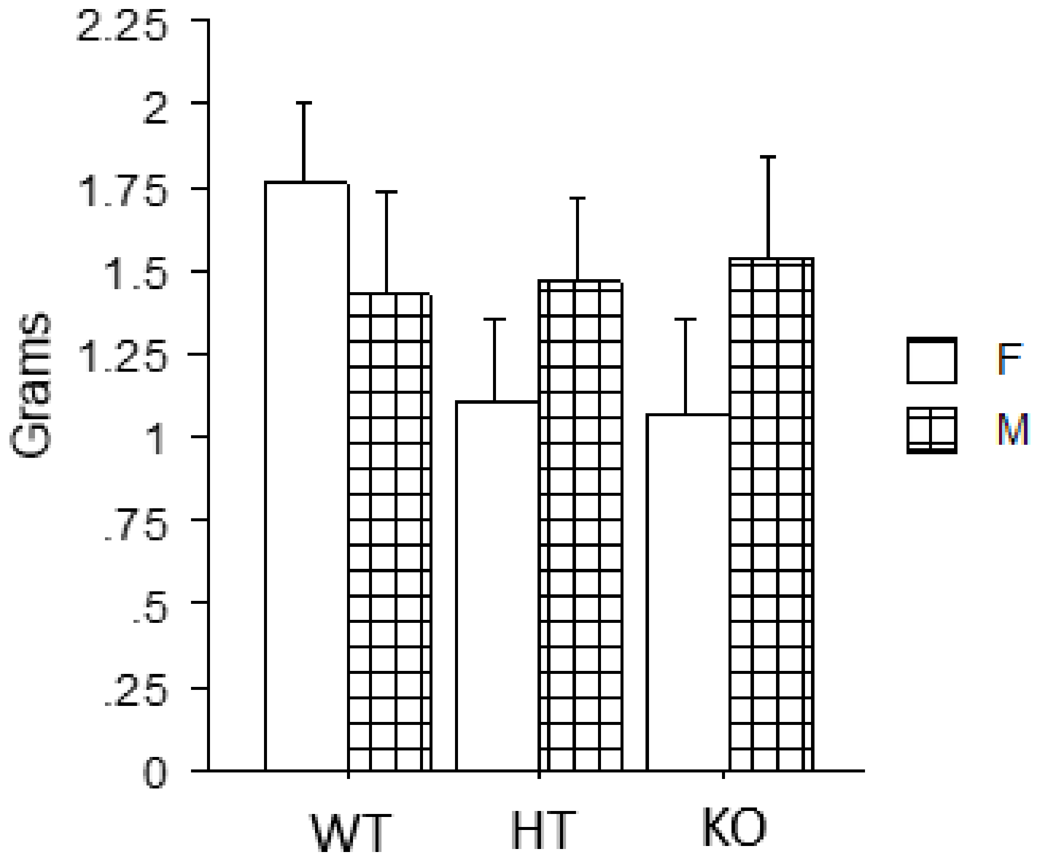

Supp. Figure 1. No effect of genotype on nest building.

Supplemental Figure 2
Prepulse Inhibition

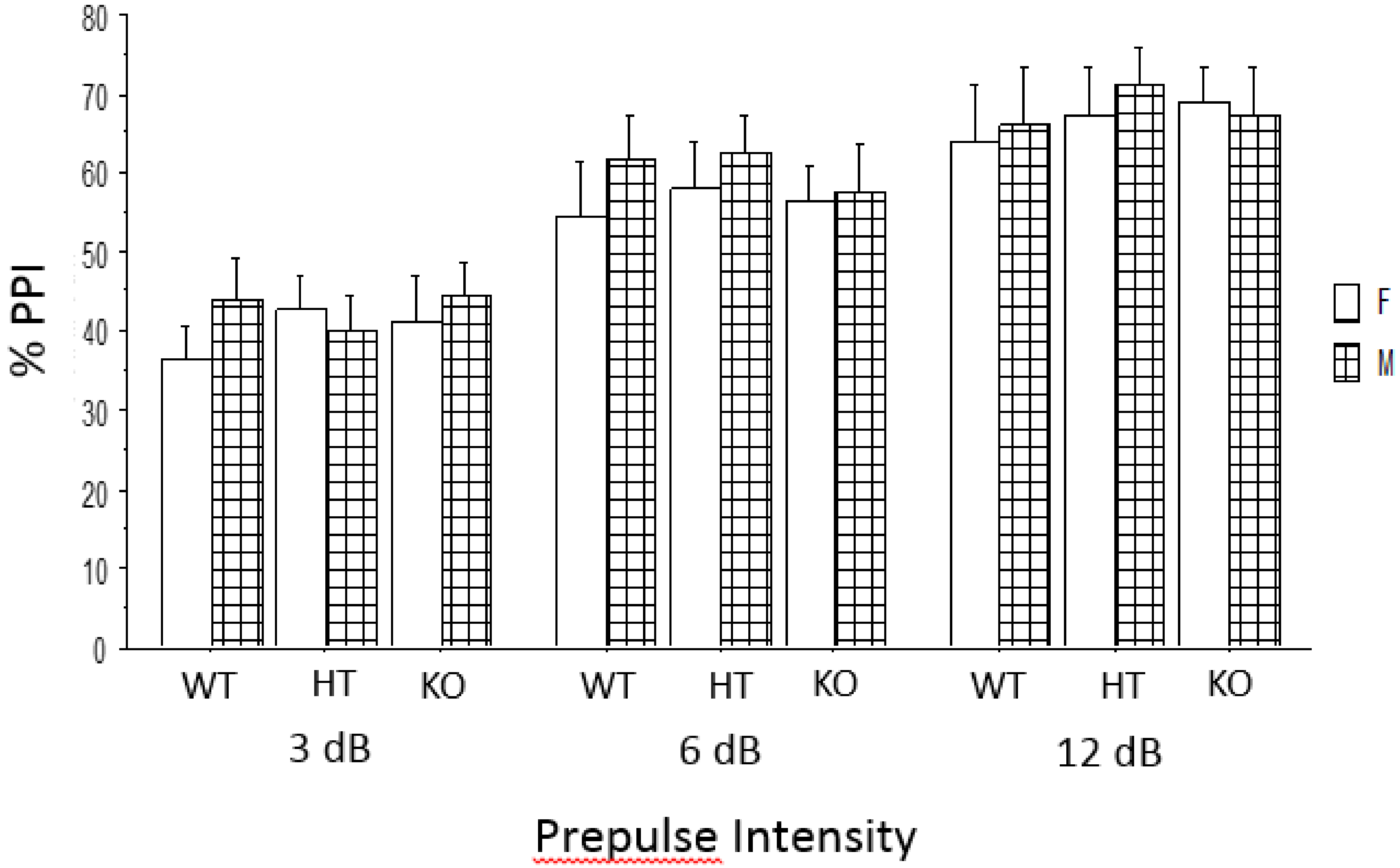

Supp. Figure 2. No effect of genotype on prepulse inhibition.

Supplemental Figure 3

A) FST: Immobility

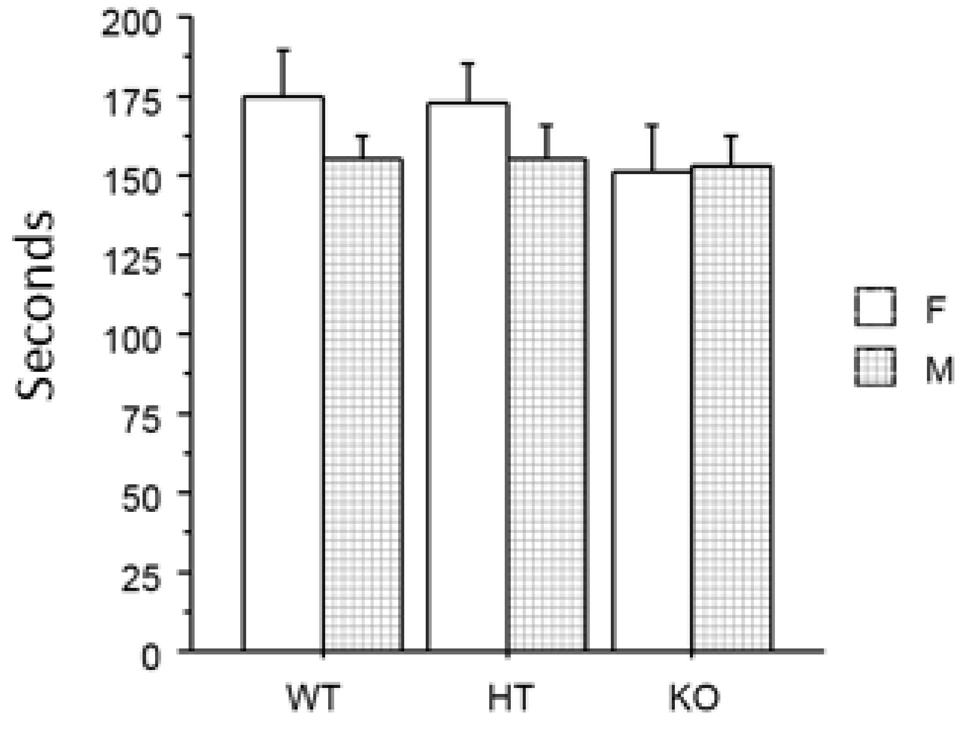

B) FST: Climbing

main effect of sex: $p < 0.05$

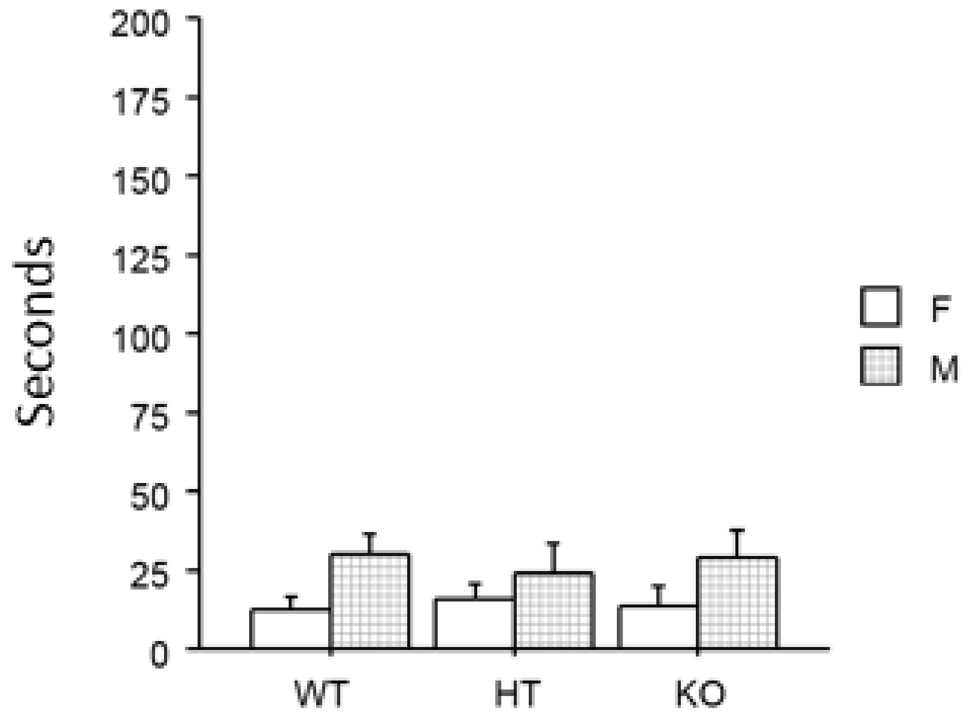

C) FST: Swimming

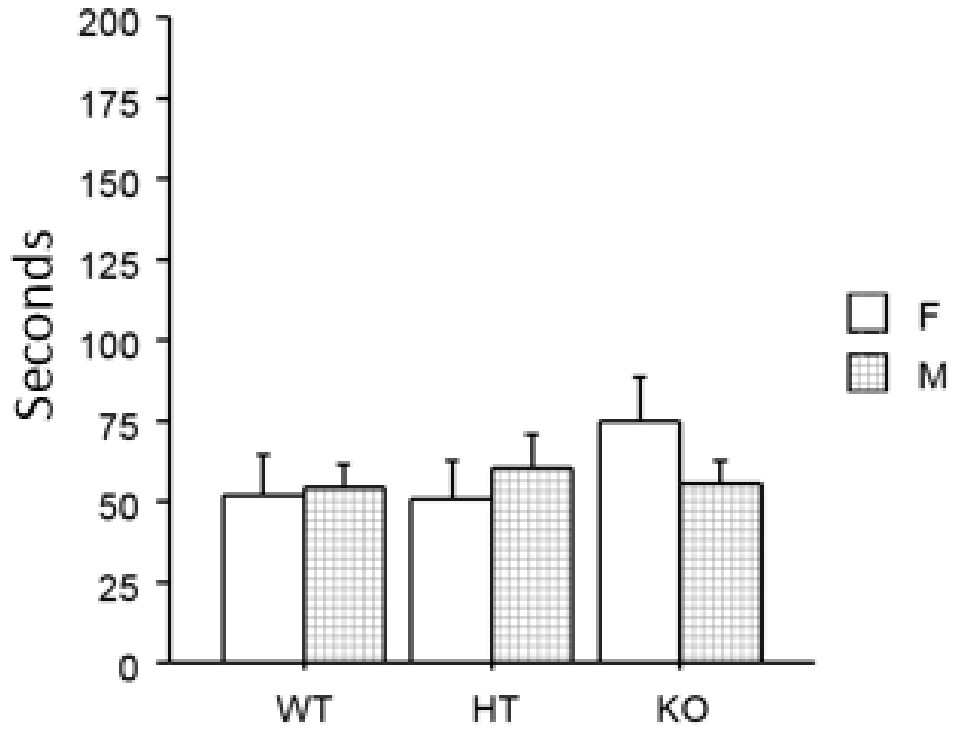

Supp. Figure 3. No effect of genotype in the forced swim test.

Supplemental Figure 4

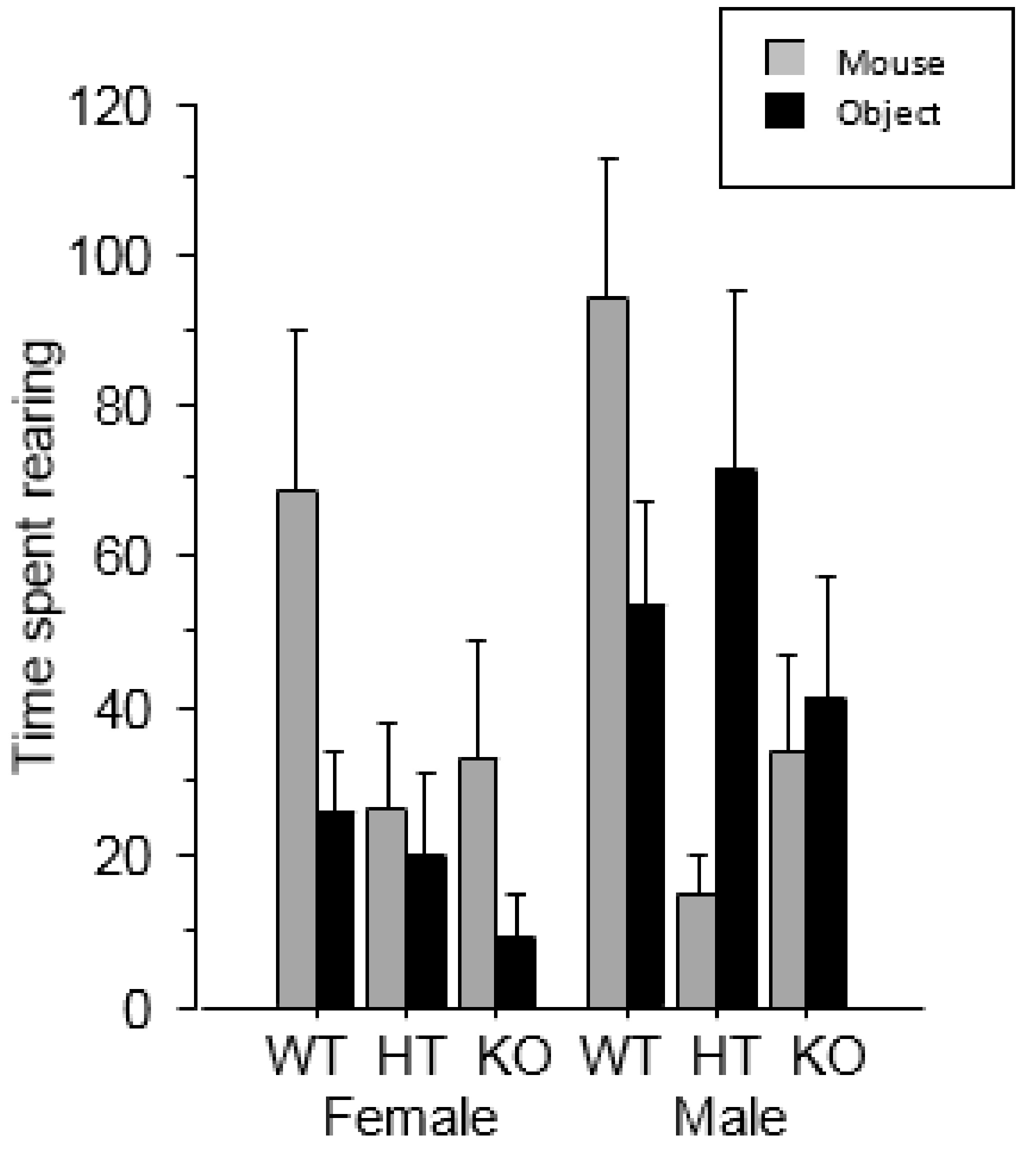

Supp. Figure 4. Time spent rearing in the zone surrounding the mouse or object.

Supplemental Figure 5
Social Interaction

Experiment 1

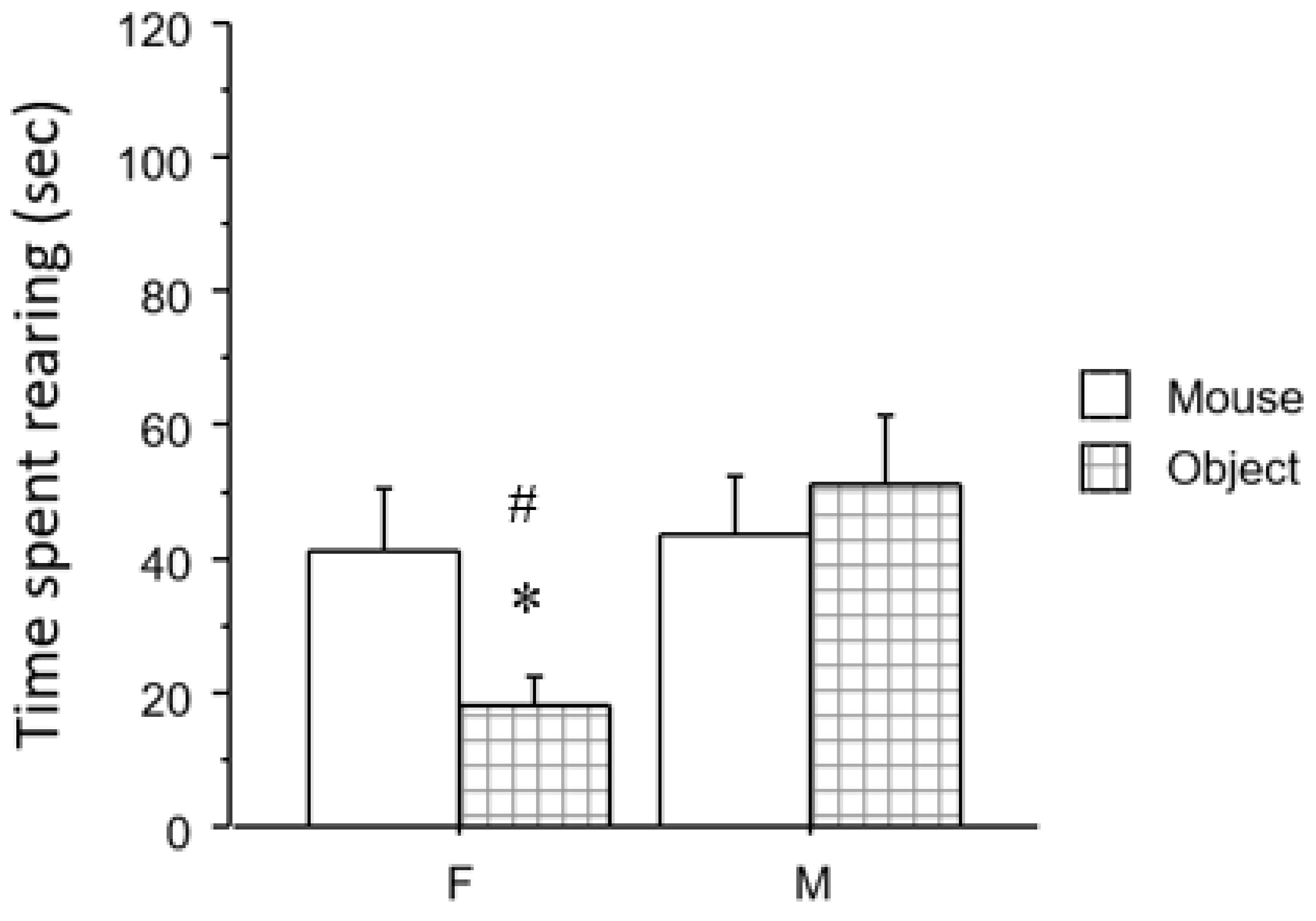

Supp. Figure 5. Female mice spent less time rearing in the zone around the object.

Supplemental Figure 6

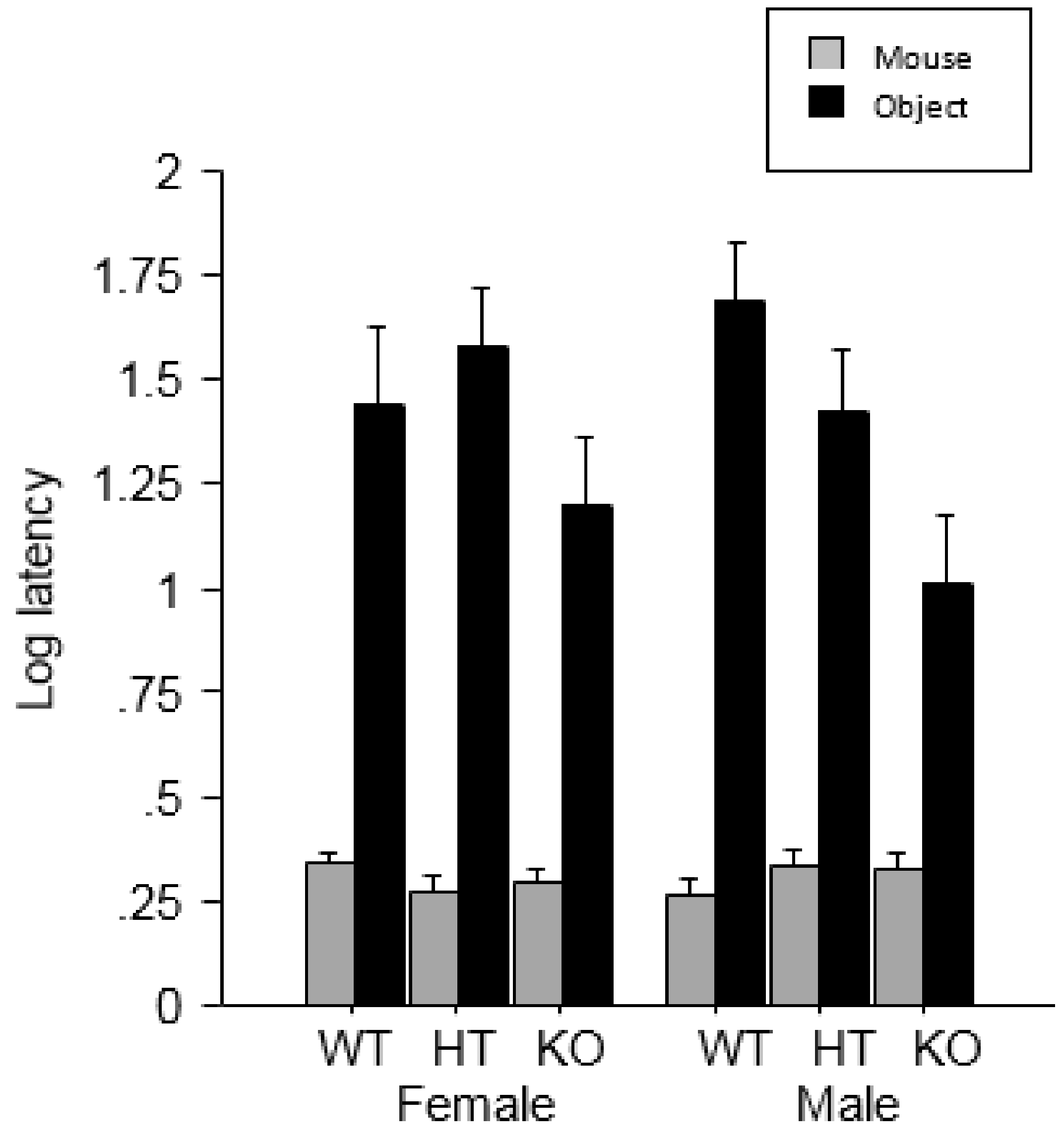

Supp. Figure 6. Log latency for mice to enter the zone around the mouse or object.

Supplemental Figure 7

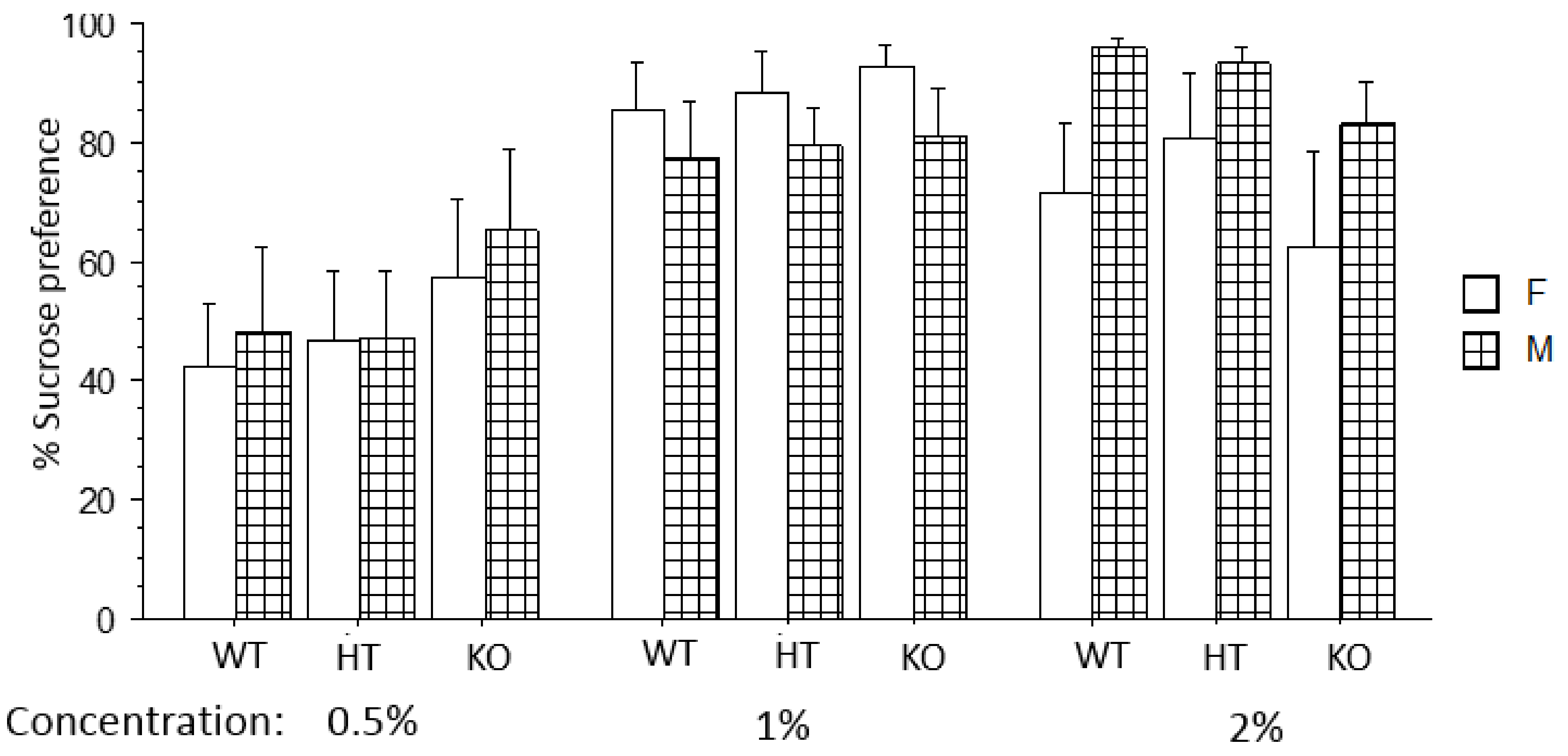

Supp. Figure 7. No effect of genotype in the sucrose preference test.
